# Data supporting characterization of CLIC1, CLIC4, CLIC5 and DmCLIC antibodies and localization of CLICs in endoplasmic reticulum of cardiomyocytes

**DOI:** 10.1016/j.dib.2016.03.061

**Published:** 2016-03-26

**Authors:** Devasena Ponnalagu, Shubha Gururaja Rao, Jason Farber, Wenyu Xin, Ahmed Tafsirul Hussain, Kajol Shah, Soichi Tanda, Mark A. Berryman, John C. Edwards, Harpreet Singh

**Affiliations:** a Department of Pharmacology and Physiology, Drexel University College of Medicine, Philadelphia, PA 19102, United States; b Department of Biological Sciences, Ohio University, Athens, OH 45701, United States; c Department of Biomedical Sciences, Ohio University, Athens, OH 45701, United States; d Division of Nephrology, St. Louis University, St. Louis, MO 63110, United States

**Keywords:** Chloride intracellular channels, Endoplasmic reticulum, Mitochondria, Cardiomyocytes

## Abstract

Chloride intracellular channel (CLICs) proteins show 60–70% sequence identity to each other, and exclusively localize to the intracellular organelle membranes and cytosol. In support of our recent publication, “Molecular identity of cardiac mitochondrial chloride intracellular channel proteins” (Ponnalagu et al., 2016) [1], it was important to characterize the specificity of different CLIC paralogs/ortholog (CLIC1, CLIC4, CLIC5 and *Dm*CLIC) antibodies used to decipher their localization in cardiac cells. In addition, localization of CLICs in the other organelles such as endoplasmic reticulum (ER) of cardiomyocytes was established. This article also provides data on the different primers used to show the relative abundance of CLIC paralogs in cardiac tissue and the specificity of the various CLIC antibodies used. We demonstrate that the predominant CLICs in the heart, namely CLIC1, CLIC4 and CLIC5, show differential distribution in endoplasmic reticulum. CLIC1 and CLIC4 both show co-localization to the endoplasmic reticulum whereas CLIC5 does not.

**Specifications Table**

**Table:** 

Subject area	*Biology*
More specific subject area	*Cardiac intracellular ion channels*
Type of data	*Table, microscopy, Western blots and text files*
How data was acquired	*Microscope (Olympus IX81), Western blotting*
Data format	*Filtered, analyzed*
Experimental factors	*Specificity of CLIC antibodies using specific knock out mice and clic*^*109*^*mutant flies.*
Experimental features	*Confocal microscopy to determine the localization of CLICs in cardiac endoplasmic reticulum, specificity of the antibody used is checked by Western blotting using specific CLIC KO mouse cardiac tissues and Drosophila mutant cardiac tubes.*
Data source location	*Laboratory animals from Charles Rivers, PA*
Data accessibility	*Data is with this article*

**Value of the data**

• Our data describing the differential localization of CLICs in cardiac cells provide a possible mechanism for their diverse functional roles in the heart.
• CLIC4 [2] and CLIC5 [3] as mitochondrial channel proteins suggest their role in maintaining mitochondrial physiology.
• CLIC1 [4] and CLIC4 [2] localization in endoplasmic reticulum might suggest their role in handling endoplasmic calcium like CLIC2, *via* modulating ryanodine receptors.
• Listed antibodies are characterized for their specificity which can be used by other investigators.

## 1 Data

Table 1 provides the list of primers used to determine the relative abundance of each CLICs in the cardiac tissue [1].

**Fig. 1:**
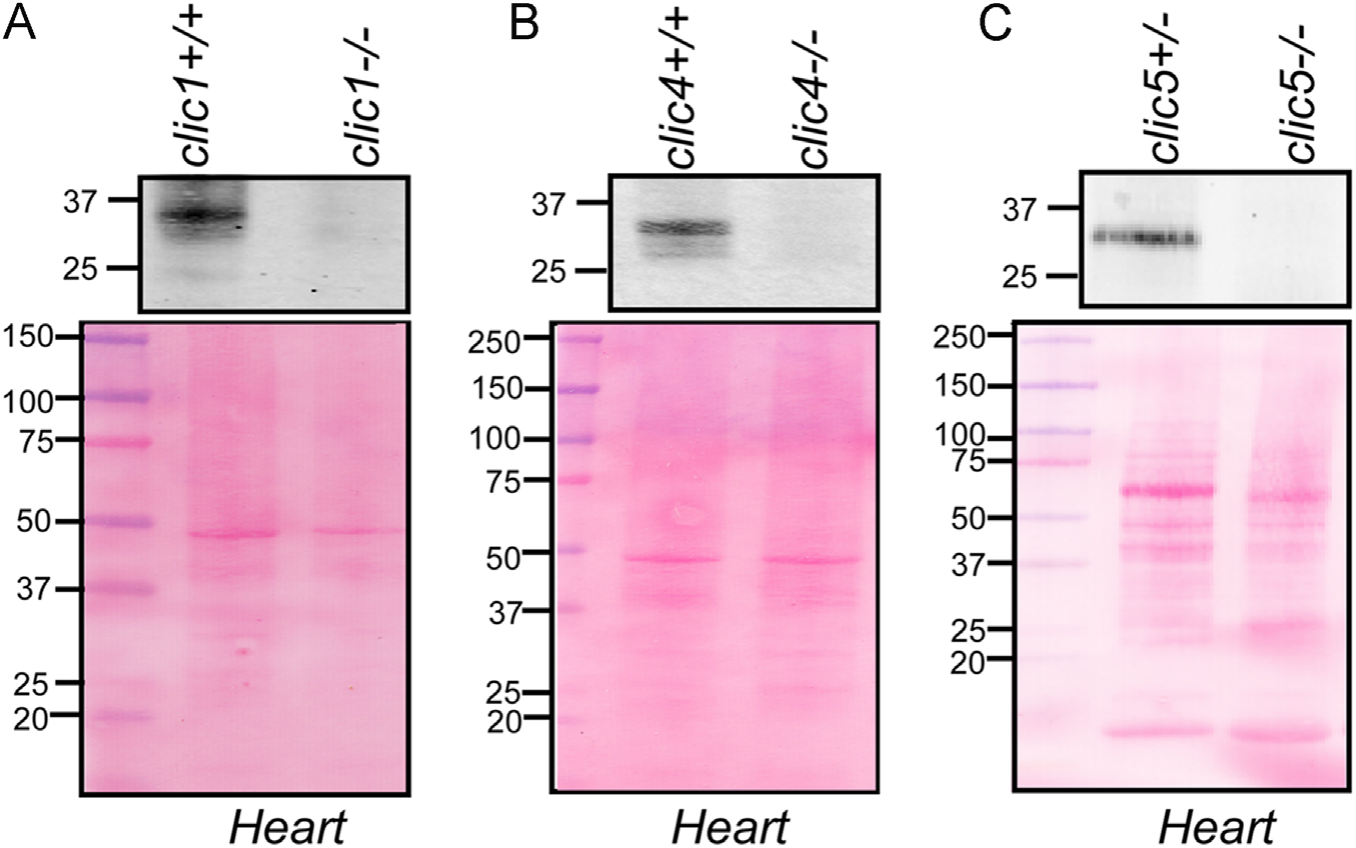
Specificity of CLIC antibodies using CLIC1, CLIC4 and CLIC5 knock out (KO) mice heart lysates**.** (I) 50 µg of heart lysates from WT, *clic1*^*−/−*^, *clic4*^*−/−*^ and *clic5*^*−/−*^ mouse were electrophoresed on 4–20% (w/v) SDS-PAGE, transferred onto nitrocellulose membrane and probed with anti-CLIC1, anti-CLIC4 and anti-CLIC5 antibodies. Absence of CLIC1, CLIC4 and CLIC5 specific bands in KO organ samples confirms the specificity of the antibody. Corresponding Ponceau S stained nitrocellulose membrane is shown as a loading control.

Specificity of the CLIC antibodies was evaluated using the cardiac tissue lysates from the *clic1*^*−/−*^*, clic4*^*−/−*^ and *clic5*^*−/−*^ mice. Absence of CLIC specific band in the knock out cardiac lysates indicated the specificity of the antibodies used in this study (Fig. 1).

**Fig. 2:**
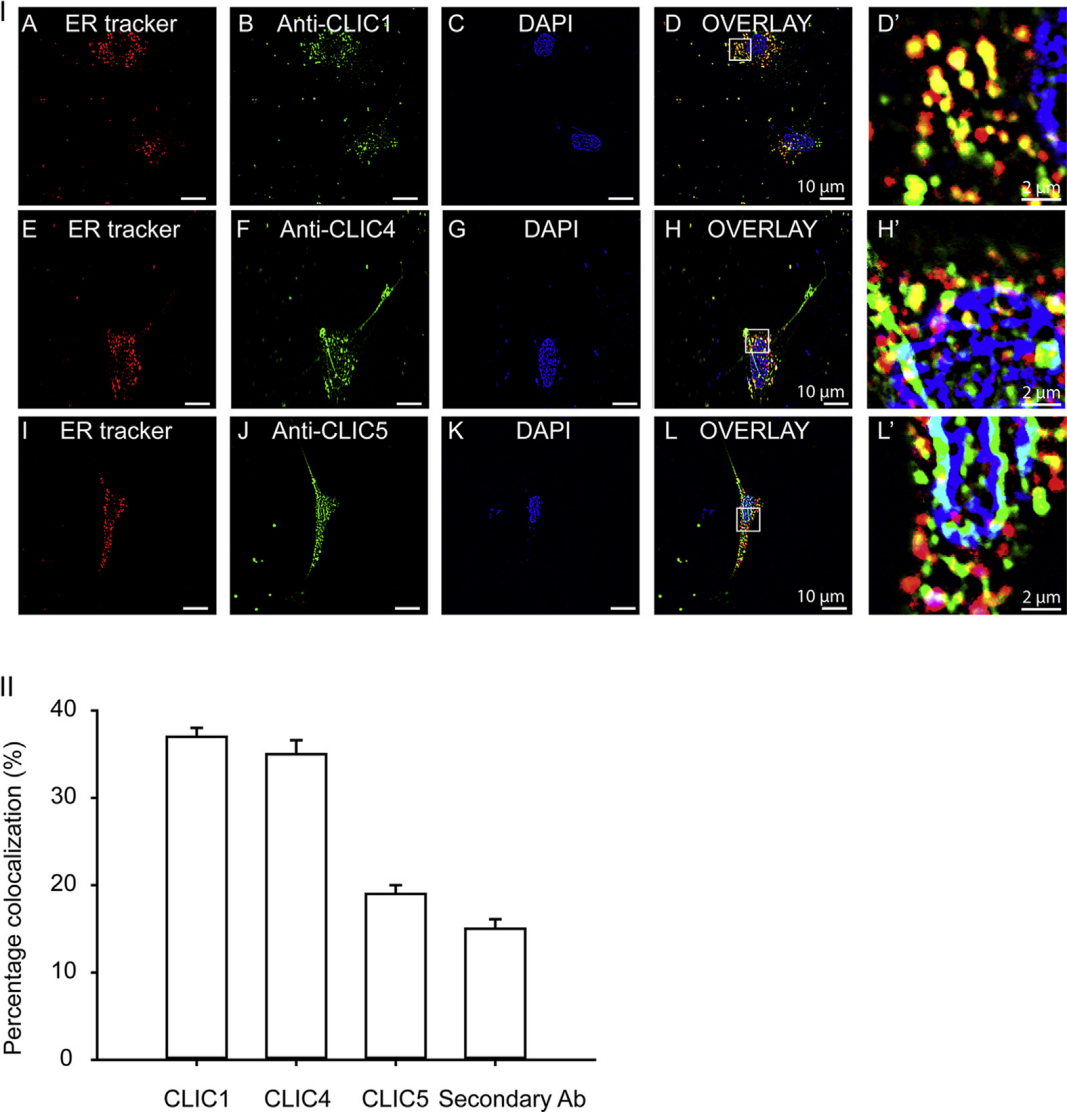
Localization of CLIC1, CLIC4 and CLIC5 to the endoplasmic reticulum. (I) Isolated p3 neonatal cardiomyocytes were loaded with ER tracker (A, E, I), fixed, permeabilized, labeled with anti-CLIC1 (B), anti-CLIC4 (F), anti-CLIC5 (J) antibodies and further stained with DAPI (C, G, K). D, H and L are merge images showing colocalization of CLIC1 and CLIC4 to the endoplasmic reticulum whereas CLIC5 shows much less colocalization. D′, H′ and L′ are enlarged images of the squared region. (II) Bar graph representing percentage colocalization of CLIC1, CLIC4 and CLIC5 to endoplasmic reticulum (*n*=3).

Localization of CLIC1, CLIC4 and CLIC5 was deciphered in endoplasmic reticulum (ER) of neonatal cardiomyocytes using the highly specific antibodies (Fig. 1). CLIC1 (37±1.0%, *n*=3) and CLIC4 (35±1.6%, *n*=3) showed higher degree of colocalization to the endoplasmic reticulum whereas CLIC5 (15±1.1%, *n*=3) showed negligible colocalization (Fig. 2).

**Fig. 3:**
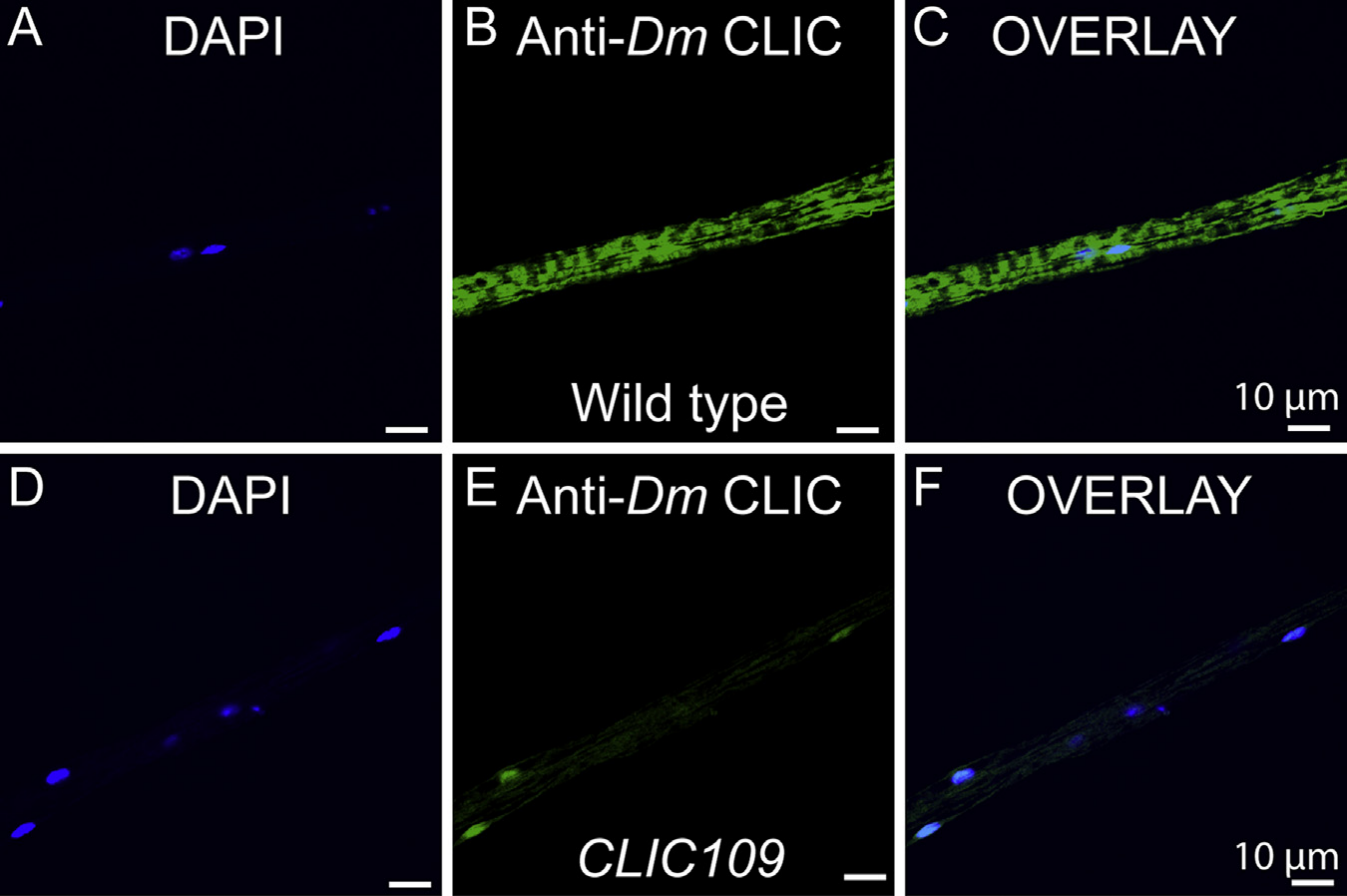
Specificity of *Dm*CLIC antibody using *clic^109^* mutant**.** Cardiac tubes of Wt *D. melanogaster* (A–C) and*clic^109^* mutant fly (D–F) were fixed, permeabilized and labeled with anti-*Dm*CLIC antibody (B, E) and further stained with DAPI (A, D). C and F are merge images. No staining for CLIC in the cardiac tubes of *clic^109^*mutant fly was observed (E, F) (*n*=3).

*Dm*CLIC, an ortholog of CLIC in *Drosophila melanogaster*, localizes to the mitochondria of cardiac tubes [1] as well. Further, specificity of the *Dm*CLIC antibodies was tested in cardiac tubes of CLIC mutant flies, *clic^109^* (Fig. 3).

## 2 Experimental design, materials and methods

The following methods support the results section of the published manuscript describing molecular identity of mitochondrial CLIC proteins.

## 3 Real time PCR analysis and quantification of various CLIC transcripts

Hearts from the 2 month old Sprague-Dawley rat were used to prepare total RNA using TRIZOL reagent (Life technologies). This was followed by double digestion with RNase-free DNase for 30 min at 37 °C. RNA prepared was further cleaned up with RNeasy mini kit (Qiagen). Purified RNA (2 μg) was reverse-transcribed with Omniscript Reverse Transcription (RT) kit (Qiagen) using oligodT primer. The reverse transcriptase was inactivated by heating at 95 °C for 5 min. Real-time qPCR was performed using iQ SYBR Green Supermix (Bio-Rad) in applied biosciences system (iQ cycler, Applied Biosystems), according to MIQE guidelines [5], [6]. 1 μl of RT reaction product, and 0. 3 µl of 25 pmole/µl primer pairs (Table 1) were added in a 20-μL reaction. Primer pairs were designed to flank an intron to control contamination from genomic DNA. The amplification conditions comprised an initial denaturation step at 95 °C for 5 min, and 40 cycles of 95 °C for 45 s, 60 °C for 45 s, and 72 °C for 45 s. Mock cDNA (no reverse transcriptase) as well as primers to amplify GAPDH were used as a control to assess the quality of the cDNA and genomic DNA contamination. GAPDH will be used to normalize the expression of different CLICs. All samples were run in triplicates. Threshold cycle values (Cq) were measured at a fluorescence of 100 a.u. Efficiency of the primers was calculated using the slope of the standard curve plot (threshold cycle *vs*. log of various DNA concentration) defined as (10–1/slope–1)×100. Clear single peaks at their melting temperature and a clear band at the expected size in agarose gels confirmed the amplification of specific products.

## 4 Immunolabeling

Isolated mitochondria and cardiomyocytes (neonatal and adult) were seeded in glass coverslips (0.17-mm thickness) coated with either poly-d-lysine or poly-l-lysine [0.1% (v/v) in PBS], respectively. After, 24 h, samples were preloaded with 200 nM mitotracker/ER tracker (as described below) and then fixed with 4% (w/v) paraformaldehyde in PBS for 10 min at room temperature followed by permeabilization with 0.5% (v/v) Triton-X 100 in PBS for 10 min at room temperature. Samples were blocked with 10% (v/v) normal goat serum (NGS; G9023; Sigma Aldrich) in 0.1% (v/v) Triton-X 100/PBS for 30 min at room temperature to control nonspecific binding. Samples were incubated overnight at 4 °C with specific antibodies diluted in PBS containing 1% (v/v) NGS and 0.1% (v/v) Triton-X 100. After washing three times with PBS containing 0.1% (v/v) Triton-X 100, samples were incubated at room temperature for 60 min with corresponding secondary antibody conjugates Atto 647N (1 μg/mL each of anti-mouse and anti-rabbit IgGs) or Alexa-488 (2 μg/mL anti-mouse IgG or anti-rabbit IgG) in 0.1% (v/v) Triton-X 100 in PBS, containing 1% (w/v) NGS. Samples were mounted for confocal microscopy with mowiol® 4-88 (Sigma Aldrich). Images were acquired with an Olympus confocal microscope IX 81 using a 60× oil immersion objective with 1.42 NA (PlanAppoN) and median filtered [6], [7]. Percentage colocalization was quantified using image J.

(1) *Neonatal cardiomyocytes:* Differentiated cardiomyocytes seeded on the poly-d-Lysine coated coverslip were loaded with 200 nM mitotracker or 200 nM ER tracker and incubated at 37 °C for 10 min and then washed with ice cold PBS. After loading the cells were fixed, permeabilized, and labeled with anti-CLIC1 [0.2 µg/mL, SC-271051 (lot no: E1711), Santa Cruz], CLIC4 [0.2 µg/mL, SC-135739 (lot no: D1911), Santa Cruz] and CLIC5 [0.2 µg/mL, ACL-025 (lot no: ANO102) Alomone lab] antibodies as mentioned above.
(2) *Adult rat cardiomyocytes:* Dissociated cardiomyocytes were immediately transferred onto poly-l-lysine coated coverslips for 1 h at 4 °C and then loaded with 200 nM mitotracker for 10 min at 37 °C. Samples were then fixed, permeabilized, and labeled with anti-CLIC1, CLIC4 and CLIC5 antibodies (0.2 µg/mL, each).
(3) *Cardiac mitochondria:* Isolated mitochondria were incubated with 200 nM mitotracker for 60 min at 4 °C on a rotator shaker. After loading, mitochondria were seeded onto poly-l-lysine coated coverslips for 2 h at 4 °C, fixed, permeabilized, and labeled with anti-CLIC1, CLIC4 and CLIC5 antibodies.
(4) *Drosophila*: Cardiac tubes of wild type as well as *Clic^109^ D. melanogaster* were isolated, fixed, permeabilized and labeled with anti-*Dm*CLIC (1:500) and anti-ATP synthase (20 ng/mL, ab14748, Abcam) antibodies.

## 5 Super resolution microscopy

STED images were acquired with a custom-made STED nano-scope using an oil immersion objective (HCX PL APO 100×/1.40-0.70 OIL CS, Leica Germany) as described earlier [8]. A 635 nm pulsed diode laser (LDH-D-C-635, PicoQuant GmbH) was used for excitation. A tunable Ti:sapphire laser (Mai Tai HP, Spectra Physics) set at 780 nm was used to deliver pulses for STED depletion. Fluorescence emission from ATTO 647N-labeled secondary antibodies was collected through a Semrock BrightLine FF01-692/40-25 nm band pass filter in front of a photomultiplier (H7422PA-40, Hamamatsu Photonics K.K.). Images (955×960 pixels) were acquired with a 16 kHz line frequency (resonant mirror of 8 kHz) and summed 256 times. Pixel size was ~9.575 nm×9.575 nm. For comparison between conventional confocal images and STED images, all imaging parameters were kept identical except for the number of summations which was 64 when recording confocal images. Confocal images were acquired first for the same field prior to STED imaging. For analysis, STED images were filtered by mean subtraction with a filter window width and height of 32 and threshold was set to 1 [9].

## 6 Sub-cellular fractionation of mitochondria

Mitochondria was sub-fractionated as described earlier [10]. Briefly, hearts from 2 months old rats were excised, washed with PBS and then homogenized in mito-isolation buffer (mmole/L, 3 HEPES-KOH, 210 mannitol, 70 sucrose, 0.2 EGTA, complete mini EDTA-free protease inhibitor cocktail) followed by centrifugation at 2500*g* for 5 min at 4 °C. The supernatant was spun again at 12,000*g* for 10 min for separating mitochondria at 4 °C. The pellet enriched with mitochondria was then resuspended in mito-isolation buffer containing 2.5 mg/mL of digitonin and then vortexed for 15 min. The suspension was then centrifuged again at 12,000*g* for 10 min at 4 °C. The supernatant containing outer mitochondrial membrane and inter-membrane space was transferred to another tube. The pellet was resuspended in 500 μl of mito-isolation buffer containing 2.5 mg/mL of digitonin and then sonicated briefly in ice-cold water sonicator. This was followed by centrifugation at 100,000*g* for 30 min at 4 °C. After centrifugation the pellet containing inner mitochondrial membrane was stored for further analysis in Western blot.

## 7 Biochemical analysis

Rat brain, heart, kidney, spleen, Percoll-purified mitochondria or sub-fractioned cardiac mitochondria samples were treated with lysis buffer [RIPA mmole/L, 50 Tris–HCl, 150 NaCl, 1 EDTA-Na_2_, 1 EGTA-Na_4_, 1 Na_3_VO_4_, 1 NaF, 1% (v/v) Nonidet P-40, 0.5% (w/v) Na-deoxycholate and 0.1% (w/v) SDS, pH 7.4] containing protease inhibitors (1 tablet/50 mL; Roche), and incubated for 1 h at 4 °C with shaking. Samples were centrifuged at 10,000*g* for 30 min and the lysates (supernatants) were collected. Similar treatment was done for CLIC1, CLIC4 and CLIC5 knock out cardiac tissue from mice. Proteins (50 μg) were separated on 4–20% (w/v) SDS/PAGE and transferred to nitrocellulose membranes (wet-transfer). Loading was corroborated with Ponceau S staining. Membranes were blocked with LICOR blocking buffer in TBS for 2 h at room temperature. Respective blots were incubated overnight at 4 °C with anti-CLIC1 mAb (0.2 μg/mL), CLIC4 mAb (0.2 μg/mL), CLIC5 pAb (0.2 µg/mL), Cox2 (0.2 µg/mL, ab15191, Abcam), GRP78Bip (1 µg/mL, ab21685, Abcam,), Lamin-B1 (0.1 µg/mL, ab16045, Abcam), GM130 (1 µg/mL, G7295 Sigma,), pan-cadherin (1 µg/mL, C8121, Sigma), ATP synthase (20 ng/mL, ab14748, Abcam,) and VDAC1 (NeuroMab, 1 µg/mL) antibodies. Membranes were washed thrice with 1X Tris-Buffered Saline containing Tween-20 and incubated with 0.01 μg/mL secondary Abs (IRdye 800 goat anti-mouse IgG and IRdye 800 goat anti rabbit IgG) for 60 min at room temperature. After extensive washing, membranes were visualized using Odyssey Imaging System (Li-Cor).

## Figures and Tables

**Table 1:** Sequence of the primers used in real time PCR.

Name	Gene ID	Forward primer (5′–3′)	Reverse primer (5′–3′)	Size	Exons
CLIC1	NM_001002807.2	CTCAGAGGAAGTTTCTGGATGG	GGATGGTGAATCCTCTGTACTTT	111	5 and 6
CLIC2	NM_001009651	CCCAAGCAGACCCTGAAAT	CTCCCTTAAGCCAGAGTATCATAAAA	111	1 and 2
CLIC3	NM_001013080	AAGCTCCAGCTCTTTGTGAAG	TGAGGAGCAGGACCATGAA	92	1 and 2
CLIC4	NM_031818.1	GACTGAAGGAGGAGGACAAAG	ATCATGAAGAGCCTCTGTGAAA	106	1 and 2
CLIC5	NM_053603.2	GCAAGGCACAGGGAATCTAA	CTCTCAAGGGCAGCATTGT	101	4 and 5
CLIC6	NM_176078	CACGACATCACCCTCTTTGT	CCCTTTAGCCAGAGGATCATAAA	98	3 and 4
