# Supplementary material for: Data supporting characterization of CLIC1, CLIC4, CLIC5 and DmCLIC antibodies and localization of CLICs in endoplasmic reticulum of cardiomyocytes
Source: Data Brief. 2016 Mar 26;7:1038–44. doi: 10.1016/j.dib.2016.03.061 (PMC4826591; doi:10.1016/j.dib.2016.03.061)
Supplement: Supplementary file 1 — Supplementary material [file mmc1.docx]

**Conflict of Interest Form**

Dear Editor,

We do not have any conflict of interest for this Data in Brief Manuscript.

Thank you,

Harpreet Singh Ph.D.

(on behalf of all authors)
